# The effect of short term peripheral parenteral nutrition on treatment outcomes and mortality in critically ill pediatric canine patients

**DOI:** 10.1186/s13620-021-00194-2

**Published:** 2021-06-03

**Authors:** Cesar Augusto Flores Dueñas, Soila Maribel Gaxiola Camacho, Martin Francisco Montaño Gómez, Rafael Villa Angulo, Idalia Enríquez Verdugo, Tomás Rentería Evangelista, José Ascención Pérez Corrales, Miguel Ángel Rodríguez Gaxiola

**Affiliations:** 1 Veterinary Medicine and Zootechnics School, Autonomous University of Sinaloa, Culiacán, Mexico; 2 Veterinary Sciences Research Institute, Autonomous University of Baja California, Mexicali, Mexico; 3 Engineering Institute, Autonomous University of Baja California, Mexicali, Mexico

**Keywords:** Peripheral parenteral nutrition, Pediatrics, Critically ill, Hospital stay, Mortality, Complications

## Abstract

**Background:**

Peripheral parenteral nutrition (PPN) is increasingly considered as an alternative to central parenteral nutrition (CPN) given the higher cost and more frequent clinical complications associated with the latter. However, the assessment of potential risks and benefits of PPN in critically ill pediatric canine patients has not been extensively performed. In this study, we aimed to explore the effect of short-term, hypocaloric PPN on weight loss, length of hospital stay, the incidence of complications, adverse effects, and mortality in critically ill pediatric canine patients.

**Results:**

Between August 2015 and August 2018, a total of 59 critically ill pediatric canine patients aged from 1 to 6 months admitted at the Veterinary Sciences Research Institute of the Autonomous University of Baja California were included in this non-randomized clinical trial. Canine pediatric patients were initially allocated to 3 groups: 11 in group 1 receiving parenteral nutrition (PN) supplementation equivalent to 40% of the resting energy requirement (RER), 12 in group 2 receiving supplementation of 50% of the RER, and 36 in group 3 receiving no PN supplementation. After establishing that there was no significant difference between 40 and 50% of PN supplementation, these groups were not separated for downstream analysis.

Similar lengths of hospital stays were noted among study subjects who received PN supplementation and those who did not (4.3 ± 1.5 vs. 5.0 ± 1.5, days, *p* = 0.097). No metabolic-, sepsis- or phlebitis-related complications were observed in any animal in the PPN supplemented group. Higher mortality (19.4% vs. 0%, *p* = 0.036), and a greater percentage of weight loss (9.24% vs. 0%, *p* <  0.001) were observed in patients who received no supplementation.

**Conclusion:**

Even though short-term, hypocaloric PPN did not reduce the length of hospital stay, it was associated with lower mortality and resulted in mitigation of weight loss. In contrast to previous studies evaluating central and peripheral parenteral nutrition protocols, we observed a lower frequency of metabolic, septic, and phlebitis complications using a 40–50% parenteral nutrition treatment. The parenteral nutrition therapeutic intervention used in our study may reduce PN-related adverse effects and promote a favorable disease outcome in critically ill canine patients. Larger studies will be needed to confirm these observations.

## Background

Nutritional supplementation is essential to the care of critically ill veterinary patients and promotes a favorable outcome [1]. Enteral nutrition or feeding through the gastrointestinal tract (GI) is the preferred route of nutrient delivery [2–4]. Early enteral nutrition decreases hospitalization times for both canine and human patients by preserving gastrointestinal function, intestinal permeability, and microbial diversity [5, 6]. However, enteral nutrition can be difficult to administer in critically ill canine pediatric patients with certain gastrointestinal disorders, especially gastric or intestinal motility disorders, which prevent effective absorption and use of essential nutrients. These veterinary patients may become malnourished early in their care, and malnutrition may increase morbidity and mortality [7].

Previous research in dogs and cats has suggested that the initiation of parenteral nutrition (PN) can reduce mortality when enteral feeding cannot be provided [8]. However, PN is also known to be associated with complications, such as septic complications, metabolic abnormalities, acid-base disturbances, hyperglycemia, hypertriglyceridemia, and hepatobiliary disorders [6, 7, 9]. Over-nutrition has been suggested as another main cause of complications of PN in human and small animal patients [10, 11], although this has not been extensively studied in small animals.

The potential effects and benefits of peripheral parenteral nutrition (PPN), which typically provides a fraction of the resting energy requirements (RER), on the condition of ill canine patients are still unclear. Despite evidence suggesting that PPN may be suitable to support the energy demand of critically ill pediatric patients for short periods of hospitalization, further clinical studies are needed to document the effects of short-term PN on the incidence of common complications and overall hospital stay in canine patients. Based on the observation that supplementation of 100% of RER (central or total parenteral nutrition) was correlated with unwanted side-effects in human and small animal patients [6, 8, 10, 11], we aimed to determine whether hypocaloric supplementation would have beneficial effects on mortality, morbidity, and hospital stay compared to treatment without PN while minimizing adverse effects.

Here we set out to test our specific hypothesis that the administration of PPN providing 40–50% of the RER would have an overall positive effect on mortality, body weight, length of hospital stay, and clinical condition of critically ill canine pediatric patients compared to those without parenteral nutrition without increasing the incidence of adverse effects reported in other clinical veterinary studies using PN.

## Methods

### Study design, setting, and participant selection

This study was a non-randomized clinical trial. Included in the study were critically ill canine pediatric patients that were admitted to the Small Animal Veterinary Teaching Hospital in the Veterinary Sciences Research Institute of the Autonomous University of Baja California between August 2015 and August 2018. The age range of patients was between 1 to 6 months. The inclusion criteria for parenteral nutrition included conditions that did not allow optimal enteral feeding of the patients, including anorexia and vomiting not responsive to antiemetic drugs.

Enteral fasting (EF) was defined as voluntarily not eating food for varying lengths of time. We considered an enteral fasting period finished when the patient 1) voluntarily accepted food, and 2) did not vomit within 12 h after voluntary ingestion of enteral nutrition. A total of 59 study subjects were initially divided into 3 groups. Group 3 was a control group that consisted of patients with the same inclusion criteria who received conventional care but whose owners had declined initiation of PPN (no PPN supplementation), while group 1 and group 2 patients were randomly assigned and received 40 and 50% of their RER from PPN, respectively. Randomization software was used for this purpose [12]. The experimental design called for the separation of groups 1 and 2 for comparison to group 3 only if significant differences were observed between groups 1 and 2. In all supplemented patients, PPN was initiated 24 h after hospitalization, as long as the patient was adequately hydrated. Patients with any degree of dehydration 24 h following hospital admission were excluded from the study. PPN was administered for 48 h in the supplemented groups. All study subjects received standard treatment for the underlying diseases that prompted their hospital admission. The discharge criterion was adequate clinical progress without the presence of vomiting or diarrhea for 12 h.

### Data collection, procedures, and measurements

Patient registration and data collection were performed using standardized worksheets and the hospital’s medical record system. Patient characteristics, including body weight, number of days of hospital stay (measured as integers), current disease, the reason for PPN, catheter type, site of placement and observed clinical complications were recorded. Complications were classified as metabolic, septic, or phlebitis related.

Metabolic complications were defined as metabolic biochemical results outside the reference range following the initiation of PPN that was not attributable to the underlying disease and was within the reference interval prior to PN administration.

Septic complications were defined as any blood infection as a result of the nutritional supplementation process and were assessed by the presence of pyrexia or a raised white cell count not attributable to the patient’s underlying disease. Peripheral catheters were reviewed at least every 12 h for signs of infection or phlebitis. Biochemical and hematological investigations were done at the time of initiation of PPN and 48 h after. Blood glucose concentrations were monitored in all patients every 24 h. All gastroenteric patients at the hospital that were included in this clinical study received Hartmann’s Solution with 2.5% dextrose from admission and until the fluid therapy was discontinued. This commercial product was not supplemented with additional glucose and is pre-prepared (PI Fluid, PISA). All puppies in the control group and the PPN group received the same solution for both hydration and maintenance. Supplemental enteral nutrition (Hill’s Prescription Diet i/d, low fat) liquefied with water was administered in all animals, which, according to the protocol of the hospital unit was provided via a syringe starting 24 h after being hospitalized and was administered every 4 h as a short-term diet. We use this enteral regimen to maintain intestinal mucosal health and reduce atrophy of the intestinal lumen cells. Feeding was not forced and subject to voluntary consumption, with a base goal of 25% of the RER each 24 h. The amount of enteral food accepted by the patient was variable, and loss due to emesis was difficult to quantify. Therefore, the amount of retained food was not accounted for.

For study subjects who received PPN, peripheral vascular access was gained through the cephalic veins. All other treatments that needed to be given intravenously were administered through a different catheter. Intravenous catheters were non-pyrogenic, radiopaque, 22 to 24 gauge in size, and made of vialon biomaterial (BD Insyte™).

PPN regimens were initiated 24 h after admission to the veterinary hospital and once hydration status was corrected. The clinical assessment of dehydration was based on skin turgor and mucous membrane evaluation 6 times per day. Each bag of PN was replaced every 24 h by aseptic techniques. Placement and maintenance of peripheral intravenous catheters, fluid therapy lines, and PN were performed in accordance with AAHA 2018 guidelines to prevent infection [13].

The partial energy requirements (PER) necessary to achieve 40 to 50% of the RER were determined using the following standard formula: PER (kcal/day) = RER x (0.40) or (0.50) for groups 1 and 2, respectively, where RER = 70 x (patient weight in kg)^0.75^.

The osmolarity and non-protein kilocalorie to nitrogen ratios of PPN solutions were calculated using predictive equations during the PPN final calculation of ingredient distribution of the mixture using software provided by the PN mixing center before its formulation (websafe2, SAFE). The osmolarity of the solution was confirmed by osmometry during the formulation process.

Once the PER for each patient was determined, the PN formulations were adjusted accordingly to comprise a caloric distribution of 60% lipids, 20% amino acids, and 20% glucose. Individualized formulations of PN specific to the patients’ weights were prepared using 20% second-generation lipids with soybean oil and medium-chain triglycerides (MCT) (Lipofundin® MCT/LCT 20%, PISA), 8.5–10% crystalline amino acids (Levamin® NORMO, PISA) and 50% dextrose solution (DX-50 solution, PISA). Underlying disease was not considered for the final PER calculation. PN dosing (determined by the above formula) and stabilization were done in a class-100 preparation area or A-classification positive pressure chamber and horizontal laminar flow hoods of a specialized mixing center under aseptic conditions. Automatic mixing was performed in a class-100,000 or D-classification cleanroom under air injection terminal filters (HEPA filters).

The weights of all the patients in the study were obtained on a daily basis. Kilograms lost from hospital admission to discharge and the percentage of initial weight lost from admission to discharge were used when evaluating weight loss.

### Data management and statistical analysis

Power analyses were conducted using methods described in [14] based on the expected variance and effect size for each pre-selected continuous variable (e.g. length of hospital stay, decrease in weight loss). Using an alpha of 0.05, it was determined that a sample size of 16 animals per group would achieve 80% power to detect the smallest anticipated relative effect size (Cohen’s d, d = 0.2) among continuous variables with a beta probability of 0.2. We used enough subjects to exceed this sample size by 20–40%. Post-hoc power calculation for categorical variables (% survival) indicated 99.8% power to resolve the observed differences between cases and controls with the sample sizes used in this study. Collected data was transferred from an Excel 2016 (Microsoft Inc., Redmond, WA) spreadsheet to STATA 14 statistical software (College Station, TX, StataCorp LP) for analysis. Categorical variables were presented as frequencies and percentages, while continuous variables were summarized as means (±standard deviations, SD) or medians (interquartile ranges, IQR) as appropriate based on their distributions. Pearson’s chi-squared and Fisher’s exact tests were used to assess associations between categorical variables. The Student’s t-test and Mann-Whitney tests were used to compare the means and medians of continuous variables, respectively. Linear regression and Pearson’s correlation coefficient (r) were used to assess linear associations and correlations, respectively, between continuous variables. A *p*-value < 0.05 was considered to indicate statistical significance.

### Ethical considerations

The study protocol was approved by the ethics committee of the Veterinary Sciences Research Institute of the Autonomous University of Baja California. Informed consent for participation in the study was obtained from the owners of the animals.

## Results

Over a 3-year period, 59 canine pediatric patients were included in this study. All 194 patients presented with severe gastroenteropathy causing diarrhea, vomiting, and anorexia. Based on the clinical characteristics and presentation of the patients, all had a presumptive diagnosis of Parvoviral Gastroenteritis. Confirmation was obtained using a rapid enzyme immunoassay SNAP Canine Parvovirus Antigen Test in 37% of the cases and with an unconfirmed diagnosis in 73% since diagnostic testing was not accepted by the owners. Twenty-five percent of the patients were positive for concurrent Toxocara canis infection. This clinical presentation did not allow optimal enteral feeding of the patients. All study subjects shared similar demographic characteristics and met the clinical criteria for the diagnosis of systemic inflammatory response syndrome (SIRS) [15, 16] (Table 1). Of those 59 patients, 23 received PN supplementation: 11 in Group 1 (40% of RER with PN) and 12 in Group 2 (50% of RER with PN). Thirty-six (36) received no PN supplementation (group 3). There was a statistically significant difference in median PPN supplement osmolarity between group 1 and group 2 (420 [332–460] vs. 582 [569–612], mOsm/L, respectively, *p* <  0.001). The non-protein kilocalorie to nitrogen ratios were 103:1 and 97:1 in groups 1 and 2, respectively. No metabolic complications were observed in the PN group according to blood results repeated 48-h after initiation of PPN supplementation and daily glucose monitoring.

**Table 1:** Canine SIRS criteria. In order to be considered to have SIRS, a patient was required to meet 2 of the 4 criteria represented in the table as described in [16]

Criteria	Value
Temperature (C**°**)	< 37.78 or > 40
Heart rate (beats/min)	> 140
Respiratory rate (breaths/min)	> 40
WBC count (WBC × 10^3^/L)	< 6 or > 19

In our initial analysis, subjects receiving PPN were divided into groups receiving 40 and 50% supplementation (group 1 vs. group 2). No significant differences in demographic or clinical characteristics were found at baseline between these groups (Table 2). Following treatment, significant differences were only observed in reticulocyte count (*p* = 0.004), glucose concentration (*p* = 0.038) and cholesterol concentration (*p* = 0.016) between group 1 and group 2 (Table 2). We determined that stratification of the overall treatment group (groups 1 and 2) was not clinically meaningful and, therefore, we did not separate these groups for comparison with the groups with no supplementation of parenteral nutrition (Control Group) in subsequent analysis. The PN group and the no-supplemented group shared similar demographic characteristics (Table 3). Statistically significant differences between the combined treatment group and the control group are summarized in Tables 3 and 4.

**Table 2:** Clinical parameters of parenteral nutrition (PN) supplemented patients before and after PN supplementation. Patients allocated to group 1 received PN supplementation equivalent to 40% of the resting energy requirement (RER), while patients in group 2 received supplementation of 50% of the RER

	Before PN			After PN			
Characteristics	Group 1, ***N*** = 11	Group 2, ***N*** = 12	***p-***value	Group 1, ***N*** = 11	Group 2, ***N*** = 12	***p***-value	Reference ranges
**Age in months**							
Mean ± SD	4.1 ± 2.1	3.5 ± 1.3	0.381				N/A
**Sex**							
Male	2 (18.2%)	4 (33.3%)	0.408				N/A
Female	9 (81.8%)	8 (66.7%)					
**Hematocrit**							
Mean ± SD	0.38 ± 0.07	0.33 ± 0.07	0.158	0.37 ± 0.07	0.32 ± 0.06	0.102	0.37–0.55 L/L
**Hemoglobin**							
Mean ± SD	126.6 ± 25	110.3 ± 22.2	0.110	123 ± 25	105 ± 25	0.101	120–180 g/L
**Erythrocytes**							
Mean ± SD	5.3 ± 1.1	5.2 ± 1.2	0.892	5.4 ± 1.4	4.8 ± 1.1	0.244	5.5–8.5 X10^12^/L
**Leukocytes**							
Median [IQR]	2.9 [1.9–8.1]	6.9 [2.6–9.1]	0.473	9.1 [6.8–14.1]	9.0 [4.8–12.4]	0.316	6.0–17.0 X10^9^/L
**Neutrophils**							
Median [IQR]	1 [0.2–2.7]	4.4 [0.5–7.03]	0.185	4.0 [3–10.1]	5.4 [2.9–5.9]	0.761	3.0–11.5 X10^9^/L
**Bands**							
Median [IQR]	0 [0–0.1]	0.1 [0–0.6]	0.340	0 [0–0.15]	0.1 [0–0.35]	0.374	0.0–0.3 X10^9^/L
**Lymphocytes**							
Median [IQR]	1.9 [1.1–3.1]	1.3 [0.7–2.8]	0.309	2.3 [2–4.7]	2.9 [1.7–5.1]	0.517	1.0–4.8 X10^9^/L
**Monocytes**							
Median [IQR]	0.16 [0–0.6]	0.15 [0.0–0.3]	0.802	0.3 [0.1–1.0]	0.1 [0–0.575]	0.172	0.1–1.4 X10^9^/L
**Eosinophils**							
Median [IQR]	0 [0–0.1]	0 [0–0]	0.208	0 [0–0]	0 [0–0]	0.506	0.0–0.9 X10^9^/L
**Basophils**							
Median [IQR]	0 [0–0]	0 [0–0]	–	0 [0–0]	0 [0–0]	–	0.0 X10^9^/L
**Platelets**							
Mean ± SD	325 ± 134.5	233.3 ± 139.9	0.140	313 ± 125	251 ± 113	0.241	200–600 X10^9^/L
**Reticulocytes**							
Median [IQR]	0 [0–0]	10 [4–21]	0.025	0 [0–0]	17 [4–28.5]	**0.016**	< 60 X10^9^/L
**Total Solids**							
Mean ± SD	51 ± 10.5	46.83 ± 11.8	0.382	45 ± 6.8	44 ± 7.1	0.808	N/A g/L
**Glucose**							
Mean ± SD	6.2 ± 1.1	5.4 ± 1.2	0.085	5.9 ± 1.1	4.9 ± 1.1	**0.038**	4.11–7.94 mmol/L
**Urea**							
Median [IQR]	3 [2.1–4.8]	3.7 [2.48–8.15]	0.524	4.4 [2.5–8.6]	3.4 [2.9–4.9]	0.134	2.5–9.6 mmol/L
**Creatinine**							
Median [IQR]	38 [26–44]	42 [25–51]	0.735	49 [28–68]	49 [36–66]	0.771	44–159 μmol/L
**Cholesterol**							
Median [IQR]	5.0 [4.4–8.5]	4.5 [3.4–5.6]	0.078	4.8 [4.3–6.8]	4.1 [3.5–4.5]	0.058	2.84–8.27 mmol/L
**Bilirubin**							
Median [IQR]	2.0 [1–7]	3.0 [1–4.5]	0.369	3 [1–13]	2 [1–6.5]	0.251	0–15 μmol/L
**ALT**							
Median [IQR]	28 [19–40]	49 [31–81]	0.201	28 [20–44]	38 [14–72]	0.484	< 100 U/L
**AST**							
Median [IQR]	19 [14–48]	28.5 [17.75–44]	0.487	21 [19–52]	33 [14–47]	0.535	< 43 U/L
**Alkaline Phosphatase**							
Median [IQR]	324 [174–651]	348 [173–649]	0.865	300 [201–532]	372 [130–585]	0.622	N/A U/L
**Sodium**							
Median [IQR]	137 [129–145]	133 [125–140]	0.198	140 [129–143]	141 [137–144]	0.422	142–153 mmol/L
**Chloride**							
Median [IQR]	101 [99–113]	104 [93–108]	0.459	107 [96–111]	108 [100–112]	0.545	110–118 mmol/L
**Strong ion difference (SID)**							
Mean ± SD	33.3 ± 5.0	28 ± 8.7	0.220	32.3 ± 4.2	26.1 ± 8.0	**0.026**	30–40 Units
**Amylase**							
Median [IQR]	631[384–1774]	580 [315–1533]	0.587	700 [515–1175]	661 [515–1293]	0.603	< 1500 U/L
**CK**							
Median [IQR]	287 [231–629]	233 [123–339]	0.200	306 [226–450]	326 [203–441]	0.752	< 250 U/L
**Triglycerides**							
Median [IQR]	0.84 [0.6–1.13]	0.93 [0.49–1.2]	0.782	0.85 [0.6–1.14]	0.82 [0.65–1.13]	0.560	< 2.19 mmol/L
**Protein**							
Mean ± SD	45.6 ± 11	42.8 ± 10.1	0.521	43 ± 7.5	39 ± 7.6	0.222	52–82 g/L
**Albumin**							
Mean ± SD	23.6 ± 5.9	22.3 ± 5.9	0.629	22 ± 4.3	20 ± 3.4	0.399	23–40 g/L
**Globulins**							
Mean ± SD	22.3 ± 5.6	20.5 ± 6.3	0.487	21 ± 4.7	18 ± 6.1	0.220	25–45 g/L
**Potassium**							
Mean ± SD	4.5 ± 0.6	4.2 ± 0.6	0.209	4.4 ± 0.43	4.4 ± 0.58	0.780	4.2–5.6 mmol/L
**Osmolarity**							
Mean ± SD	278 ± 25	270 ± 19	0.463	278 ± 16	278 ± 13	1.00	280–330 mmol/L
**Phosphorus**							
Mean ± SD	1.8 ± 0.45	1.8 ± 0.57	0.970	2.0 ± 0.39	1.9 ± 0.46	0.58	0.8–2.0 mmol/L
**Calcium**							
Mean ± SD	2.2 ± 0.25	2.8 ± 0.6	0.04	2.17 ± 0.13	2.43 ± 0.29	0.05	2.20–2.70 mmol/L

*ALT* Alanine aminotransferase, *AST* Aspartate Transaminase

**Table 3:** Clinical parameters before and after PN supplementation. Patients in the PN group received PN supplementation equivalent to 40% or 50% of the resting energy requirement (RER), while patients in the Control Group did not receive supplementation

	Before PN			After PN			
Characteristics	PN Group PN, ***N*** = 23	Control Group Control, ***N*** = 36	***p*** -value	PN Group PN, ***N*** = 23	Control Group Control, ***N*** = 36	***p*** -value	Reference ranges
**Age in months**							
Mean ± SD	3.8 ± 1.7	4.1 ± 1.2	0.508				N/A
**Sex**							
Male	6 (26.1%)	15 (41.7%)	0.223				N/A
Female	17 (73.9%)	21 (58.3%)					
**Hematocrit**							
Mean ± SD	0.35 ± 0.07	0.33 ± 0.05	0.204	0.34 ± 0.07	0.32 ± 0.05	0.237	0.37–0.55 L/L
**Hemoglobin**							
Mean ± SD	118.1 ± 24.3	109.7 ± 19.0	0.909	113.6 ± 26	108 ± 19.3	0.572	120–180 g/L
**Erythrocytes**							
Mean ± SD	5.3 ± 1.1	5.8 ± 1.1	0.0856	5.1 ± 1.3	5.6 ± 1.2	0.139	5.5–8.5 X1012/L
**Leukocytes**							
Median [IQR]	5.8 [1.9–8.9]	10.3 [6.0–14.4]	0.014	9.1 [5.3–12.4]	10.3 [5.6–15.2]	0.148	6.0–17.0 X10^9^/L
**Neutrophils**							
Median [IQR]	2.5 [0.5–6.0]	6.9 [4.1–9.9]	< 0.001	5 [3–6]	7 [4.2–10.5]	**0.021**	3.0–11.5 X10^9^/L
**Bands**							
Median [IQR]	0 [0–0.3]	0.15 [0–1.38]	0.084	0 [0–0.3]	0.3 [0–1.2]	**0.015**	0.0–0.3 X10^9^/L
**Lymphocytes**							
Median [IQR]	1.74 [0.8–2.9]	2 [1.1–3.5]	0.222	2.4 [1.8–4.7]	2.1 [1.1–3.2]	0.086	1.0–4.8 X10^9^/L
**Monocytes**							
Median [IQR]	0.16 [0–0.5]	0 [0–0]	< 0.001	0.2 [0–0.7]	0 [0–0]	**< 0.001**	0.1–1.4 X10^9^/L
**Eosinophils**							
Median [IQR]	0 [0–0]	0 [0–0.08]	0.192	0 [0–0]	0 [0–0]	0.629	0.0–0.9 X10^9^/L
**Basophils**							
Median [IQR]	0 [0–0]	0 [0–0]	0.424	0 [0–0]	0 [0–0]	–	0.0 X10^9^/L
**Platelets**							
Mean ± SD	281.6 ± 141.7	268 ± 109.6	0.706	277.9 ± 119.1	283.1 ± 112.8	0.870	200–600 X10^9^/L
**Reticulocytes**							
Median [IQR]	0 [0–11.5]	–	–	0 [0–16]	–	–	< 60 X10^9^/L
**Total Solids**							
mean ± SD	48.8 ± 11.1	47.6 ± 5.0	0.254	44.2 ± 6.8	38.9 ± 5.4	0.123	49–68 g/L

**Table 4:** Primary outcome measures compared between PN group and Control Group. Fisher exact test was used to compare mortality between groups

Characteristics	PN Group PN, ***N*** = 23	Control Group Control, ***N*** = 36	***p*** -value
**Hospital stay, days,** ***N*** **= 58**			
Mean ± SD	4.3 ± 1.5	5.0 ± 1.5	0.097
**Mortality**			
Yes	0 (0%)	7 (19.4%)	**0.036**
No	23 (100%)	29 (80.6%)	
**Percentage of weight loss**			
Median [IQR]	0% [0–2.56]	9.24% [3.25–12.25]	**< 0.001**
**Weight loss, Kg**			
Median [IQR]	0 [0–0.2]	0.4 [0.2–0.55]	**< 0.001**

### Clinical outcome of canine patients under parenteral peripheral nutrition compared to patients who received no supplementation

Similar lengths of hospital stay were noted between study subjects who received PN supplementation (PN Group) and those who did not (Control Group) (4.3 ± 1.5 vs. 5.0 ± 1.5, days, respectively, *p* = 0.097) (Table 4). Conversely, higher mortality was observed among canine patients who received no supplementation (19.4% vs. 0%, *p* = 0.036) (Table 4). The odds ratio for mortality in the control group compared to the PN group was 11.950, [95% CI: 0.649–220.140],

No patients were euthanized in this study. A greater percentage of weight loss was observed in patients who received no supplementation (9.24% vs. 0%, *p* <  0.001) (Table 4). While at-home fasting times prior to hospitalization reported by owners were greater in the control group, there was no difference in the total enteral fasting times (in-home enteral fasting time + in-hospital enteral fasting time) between study subjects who received PN supplementation and those who did not (group 3) (6.3 ± 1.6 vs. 6.1 ± 1.4, days, respectively, *p* = 0.684) (Table 5).

**Table 5:** A summary durations of at-home fasting (as reported by the owner at the time of admission), in-hospital enteral fasting, and total enteral fasting

Characteristics	PN Group PN, ***N*** = 23	Control Group Control, ***N*** = 36	***p*** -value
**In-home enteral fasting (days)**			
Mean ± SD	2.0 ± 0.7	1.0 ± 0.7	< 0.001
**In-hospital enteral fasting**			
Mean ± SD	4.3 ± 1.5	5.0 ± 1.5	0.053
**Total enteral fasting**			
Mean ± SD	6.3 ± 1.6	6.1 ± 1.4	0.684

## Discussion

In this study, we set out to investigate the potential risks and beneficial effects of short-term hypocaloric PPN on critically ill canine patient weight, mortality, length of hospital stay, and incidence of metabolic, septic, or phlebitis-related complications. The major findings of this study were that hypocaloric PPN at 40–50% of RER resulted in mitigation of weight loss and lower mortality and was associated with no septic or metabolic complications. The lack of metabolic or septic complications with PPN was in contrast to those reported in other similar studies [8, 17–19]. We did not find any laboratory value in the treatment group in which the value was within the reference interval prior to PN administration and abnormal after its implementation. The findings of this study support evidence from a previous report [17] that suggested that a nutritional approach providing 40–70% of the RER can be an appropriate short-term therapy in dogs. The feasibility and benefits of hypocaloric parenteral nutritional support have been highlighted in this previous work, and our studies extend those findings by demonstrating the beneficial effects of PPN that is limited to 40 to 50% of RER.

Most of the recent studies on canine patients establish hyperglycemia as the most common metabolic complication associated with the administration of PN [8, 17–19]. Additionally, in human clinical studies, duration of total parenteral nutrition (TPN) therapy and high dextrose delivery were found to be important risk factors and independent predictors for developing hyperglycemia in TPN patients. The risk of hyperglycemia was predicted to increase by 10% with each additional day of TPN [20, 21].

In our clinical study, hyperglycemia was not observed as a complication of PN supplementation in our patients. In our study, only 20% of the daily kilocalories provided by parenteral nutrition came from dextrose, allowing maintenance of a glucose infusion range below 1 mg/kg of weight per minute compared to previous studies evaluating premixed PPN regimens at around 4 mg/kg/minute [17]. This may have contributed to the lack of hyperglycemic cases post-PPN supplementation in the current study. However, transient hyperglycemia may have been missed since glucose was measured once daily while on PN.

Clinical evidence in human patients suggests that increased parenteral caloric intake is an independent risk factor for septic complications in patients receiving PN [22]. Although septic complications of PN were reported in several canine studies [8, 17, 19, 21, 23], no septic complications were observed in the present study. Asymptomatic cases of sepsis or clinically silent sepsis could not be ruled since cultures were not performed. Despite a lack of clinically evident septic complications in this study, we strongly recommend strict aseptic techniques when placing and maintaining the PN administration line and when compounding the PN solution regardless of the placement site. It is our opinion the application of aseptic procedures and the use of hypocaloric PPN may be related to the low incidence of indicators of septic complications in patients supplemented with 40 and 50% of the RER in the present study.

This study demonstrated lower mortality in patients receiving hypocaloric PPN than those not receiving PPN. While guidelines favor enteral nutrition, early administration of enteral nutrition is not always possible. PN can decrease the risk of death when early enteral nutrition cannot be initiated [24]. Our findings are in line with a previous study in dogs that demonstrated that energy supply, even if modest and close to resting energy requirements appears to be positively associated with hospital discharge [1]. Further investigation regarding these specific factors is justified. The mortality in our study coincides with the mortality observed in other studies that analyze the behavior of viral gastroenteritis in hospitalized puppies in which mortalities of 10 to 20% were observed [25, 26]. A recent study that reports mortality of 19.4% in patients with viral gastroenteritis established that dogs that met the SIRS criteria on admission were about 4 times more likely to die than those who did not. Higher mortality in patients without vaccination was also observed in this study [26]. In our hospital setting, as in the current study, it is very common for owners to present their pets in advanced stages of disease with severe dehydration and marked hemodynamic repercussions. All patients included in this study were unvaccinated puppies, met the canine clinical SIRS criteria, and presented macro indicators of hypotension upon admission.

In the current study, a greater percentage of weight loss was observed in patients who received no supplementation (9.24% vs. 0%, *p* <  0.001). We have shown that although weight and nutritional health are likely not sufficiently maintained by these levels for longer durations, an intermediate level of supplementation (40–50% RER) is sufficient to maintain weight or reduce weight loss in the short term. This is parallel to a clinical study of permissive underfeeding in humans that showed that hypocaloric nutritional regimens decrease weight loss at levels similar to normocaloric regimens in the short term [11]. A limitation of using bodyweight as a readout is that this measure alone is not a reliable parameter for evaluating the efficacy of nutritional support since bodyweight usually shows considerable acute variation due to gain or loss of water. Future research should include the measurement of more exact and advanced objective parameters in the measurement of body mass and nutritional efficacy.

We recognize certain limitations in our study. While we carefully reviewed the available data on all 59 patients to ensure the homogeneity among groups for unbiased comparison, we were unable to establish a severity index across all patients as some owners did not approve all tests. This may have led to differences in the severity of disease between groups. The owner’s determination of whether PPN was administered may have also introduced selection bias to this study. Those with animals with more or less severe illness may have opted out of PPN feeding, potentially confounding mortality differences between groups. However, the inclusion criteria were the same between the PN group and Control group and we found that all patients shared similar demographic and clinical characteristics prior to any treatment (Table 3), arguing for homogeneity across the groups. Monocyte counts were lower in the control group and leukocytes and neutrophils were marginally and non-significantly higher. This may indicate some differences in the etiology of disease between groups, although these differences were small and not likely to indicate significant differences in disease severity. Enteral nutrition through nasogastric and nasoesophageal tubes is a common technique currently used in patients with severe gastropathy that has been shown to result in favorable outcomes in patients in other studies. The fact that we did not use this therapeutic technique in the patients in this investigation could be a limitation of this study.

## Conclusion

This study demonstrates that although short-term hypocaloric PPN did not reduce the length of hospital stay, it was associated with lower mortality, lower percentage weight loss, and fewer septic and PPN-related complications in critically ill pediatric canine patients. The hypocaloric PN administered in this study was safe and associated with a more favorable disease outcome than with no PN. Larger studies may refine these observations. This study does not claim to provide evidence on the long-term effects of PPN on critically ill veterinary patients, which should be a subject of further investigations.

## Data Availability

The datasets used and/or analyzed during the current study are available from the corresponding author on reasonable request.

## Abbreviations

CPN: Central Parenteral Nutrition
IQR: Interquartile Range
PER: Partial Energy Requirements
PN: Parenteral Nutrition
PPN: Peripheral Parenteral Nutrition
RER: Resting Energy Requirements
SD: Standard Deviation
TPN: Total Parenteral Nutrition
